# Bone formation rather than inflammation reflects Ankylosing Spondylitis activity on PET-CT: a pilot study

**DOI:** 10.1186/ar3792

**Published:** 2012-04-02

**Authors:** Stefan TG Bruijnen, Mignon AC van der Weijden, Joannes P Klein, Otto S Hoekstra, Ronald Boellaard, J Christiaan van Denderen, Ben AC Dijkmans, Alexandre E Voskuyl, Irene E van der Horst-Bruinsma, Conny J van der Laken

**Affiliations:** 1 Department of Rheumatology, VU University Medical Center, De Boelelaan 1117, Amsterdam, 1081 HV, The Netherlands; 2 Jan van Breemen Research Institute, Dr. Jan van Breemenstraat 2, Amsterdam, 1056 AB, The Netherlands; 3 Department of Radiology, VU University Medical Center, De Boelelaan 1117, Amsterdam, 1081 HV, The Netherlands; 4 Department of Nuclear Medicine & PET Research, VU University Medical Center, De Boelelaan 1117, Amsterdam, 1081 HV, The Netherlands

## Abstract

**Introduction:**

Positron Emission Tomography - Computer Tomography (PET-CT) is an interesting imaging technique to visualize Ankylosing Spondylitis (AS) activity using specific PET tracers. Previous studies have shown that the PET tracers [^18^F]FDG and [^11^C](*R)*PK11195 can target inflammation (synovitis) in rheumatoid arthritis (RA) and may therefore be useful in AS. Another interesting tracer for AS is [^18^F]Fluoride, which targets bone formation. In a pilot setting, the potential of PET-CT in imaging AS activity was tested using different tracers, with Magnetic Resonance Imaging (MRI) and conventional radiographs as reference.

**Methods:**

In a stepwise approach different PET tracers were investigated. First, whole body [^18^F]FDG and [^11^C](*R)*PK11195 PET-CT scans were obtained of ten AS patients fulfilling the modified New York criteria. According to the BASDAI five of these patients had low and five had high disease activity. Secondly, an extra PET-CT scan using [^18^F]Fluoride was made of two additional AS patients with high disease activity. MRI scans of the total spine and sacroiliac joints were performed, and conventional radiographs of the total spine and sacroiliac joints were available for all patients. Scans and radiographs were visually scored by two observers blinded for clinical data.

**Results:**

No increased [^18^F]FDG and [^11^C](*R*)PK11195 uptake was noticed on PET-CT scans of the first 10 patients. In contrast, MRI demonstrated a total of five bone edema lesions in three out of 10 patients. In the two additional AS patients scanned with [^18^F]Fluoride PET-CT, [^18^F]Fluoride depicted 17 regions with increased uptake in both vertebral column and sacroiliac joints. In contrast, [^18^F]FDG depicted only three lesions, with an uptake of five times lower compared to [^18^F]Fluoride, and again no [^11^C](*R)*PK11195 positive lesions were found. In these two patients, MRI detected nine lesions and six out of nine matched with the anatomical position of [^18^F]Fluoride uptake. Conventional radiographs showed structural bony changes in 11 out of 17 [^18^F]Fluoride PET positive lesions.

**Conclusions:**

Our PET-CT data suggest that AS activity is reflected by bone activity (formation) rather than inflammation. The results also show the potential value of PET-CT for imaging AS activity using the bone tracer [^18^F]Fluoride. In contrast to active RA, inflammation tracers [^18^F]FDG and [^11^C](*R*)PK11195 appeared to be less useful for AS imaging.

## Introduction

Ankylosing spondylitis (AS) is a chronic, inflammatory, rheumatic disease that usually starts at an early age and can result in irreversible bone deformation and disability in the long term. AS is characterized by inflammatory back pain, limited motion of the spine, and sacroiliitis on plain radiography. Peripheral arthritis and enthesitis may also be prominent features [1]. Patients with AS are often treated with non-steroidal anti-inflammatory drugs (NSAIDs) because they stabilize disease activity over time and, in addition, can reduce radiographic progression [2]. However, with the introduction of anti-tumor necrosis factor (anti-TNF) therapy, a more effective treatment of AS became possible. Patients with AS seem to benefit most if treatment with TNF blockers is started early in the disease course, particularly when started at a younger age [3,4]. Therefore, it is important to diagnose this disease early. Until recently, plain radiographs were obligatory for the diagnosis of AS, according to the modified New York criteria [5]. The disadvantage of this imaging technique is that it usually takes many years before the disease comes to full expression and definite radiographic sacroiliitis appears [5]. Consequently, the diagnosis is often delayed by 5 to 10 years, especially in patients with an early or incomplete clinical picture [1,6,7]. To enable earlier diagnosis, highly reliable and sensitive imaging techniques are needed.

Nowadays, magnetic resonance imaging (MRI) is believed to be a sensitive imaging modality for the detection of sacroiliitis and inflammation of the spine in early AS. MRI detects (early) inflammation by visualization of tissue edema or enhanced gadolinium contrast uptake or both. However, these imaging findings are non-specific indicators of increased free water content and increased vascularization, respectively [8,9]. Moreover, chronic AS changes, such as new bone formation in the spine (syndesmophyte formation), tend to be less well visualized on MRI than on radiographs [10]. Finally, although validated scoring methods are available, conflicting data on the sensitivity and specificity of MRI in (suspected) spondylarthropathies have been published [6,11-14]. Therefore, the precise role of MRI in visualizing disease activity of AS has not yet been fully elucidated.

Positron emission tomography (PET) is another interesting imaging technique for the diagnosis of AS. PET allows sensitive imaging of functional tissue changes (pathophysiology) in the whole body by targeting binding sites [15]. The visualization of pathophysiology makes PET potentially suitable for early detection of inflammatory processes, even before anatomical changes occur. Thereby, PET allows specificity through the use of receptor targeting tracers and allows quantification of disease activity in order to accurately monitor therapeutic effects [16]. Recently, PET-computed tomography (PET-CT) scanning was introduced as a hybrid imaging technique that combines the unique properties of sensitive imaging of pathophysiology and anatomical CT imaging as a reference [17]. In this way, PET-CT offers the opportunity to visualize (early) inflammatory changes as well as (early) structural changes such as new bone formation, which is hard to detect on MRI.

The definite pathogenesis of AS is still not clear, and different joint structures may be involved in inflammatory sites in AS [18]. Therefore, different targets for the PET tracers have to be taken into account. Synovial tissue, bone marrow, entheses, and ligaments can be affected in AS [19-21] and may need different specific tissue PET tracers. PET studies in patients with rheumatoid arthritis (RA) clearly revealed inflamed synovial tissue through the use of the glucose analogue [^18^F]-fluoro-2-deoxy-D-glucose ([^18^F]FDG), visualizing increased metabolism in synovial tissue [16,22,23], and the macrophage tracer PK11195 [(*R*)-1-(2-chlorophenyl)-N-methyl-N(1-methyl-propyl)-3-isoquinoline carboxamide] ([^11^C](*R*)PK11195) [24]. [^11^C](*R*)PK11195 binds with high affinity to peripheral benzodiazepine receptors, which are expressed mainly on macrophages [25]. Since macrophage-rich inflammation has been demonstrated in patients with AS [26], both [^18^F]FDG and [^11^C](*R*)PK11195 may be interesting tracers for detection of disease activity of AS. In addition to inflammation tracers, the bone tracer [^18^F]fluoride may have potential for AS imaging since AS is characterized by syndesmophyte formation and ankylosis in vertebral column and sacroiliac (SI) joints. [^18^F]Fluoride uptake in active bone reflects local blood flow and regional osteoblastic activity [27,28]. Indeed, [^18^F]fluoride appeared to be a potential tracer for imaging of active bone sites in a small group of patients with AS [29].

The objective of this pilot study was to investigate the potential of PET-CT for imaging AS activity by the investigation of three different tracers in a stepwise approach with MRI and conventional radiographs as references for PET-CT data. Inflammation tracers [^18^F]FDG and [^11^C](*R*)PK11195 were studied in patients with AS with low and high disease activity, and these inflammation tracers were compared with the bone tracer [^18^F]fluoride in additional patients with high disease activity.

## Materials and methods

### Patients

Twelve patients who had AS (at least 18 to not more than 70 years) and who fulfilled the modified New York criteria [5] were included between March 2008 and December 2010. Patients were excluded if they were pregnant or breast-feeding, had a pacemaker, had a creatinine clearance of less than 30 mL/minute, or had any other treatment with investigational drugs within the previous 3 months. Additionally, benzodiazepines were discontinued at least 10 days prior to inclusion to prevent (partial) blockade of benzodiazepine receptors, relevant for [^11^C](*R*)PK11195 binding. NSAIDs were continued if used at inclusion.

Five patients had low disease activity - Bath Ankylosing Spondylitis Disease Activity Index (BASDAI) of less than 4 - and seven patients high disease activity (BASDAI of at least 4). Patients with high disease activity were candidates for treatment with a TNF-blocking agent and were included before anti-TNF treatment was started. The study protocol was approved by the medical ethics committee. All patients gave written informed consent prior to participation in the study.

### Study design

In a stepwise investigation of different PET tracers, we studied the potential of PET-CT in AS by using the inflammation tracers [^18^F]FDG and [^11^C](*R*)PK11195 in patients with AS with low (*n *= 5) or high (*n *= 5) disease activity and subsequently compared [^18^F]FDG and [^11^C](*R*)PK11195 with the bone tracer [^18^F]fluoride in two patients with high disease activity (*n *= 2). As references for PET-CT data, MRI scans of the total spine and SI joints were obtained within a mean of 5 days (range of 0 of 17 days) after the PET-CT scan. Conventional radiographs of the total spine (anterior-posterior and lateral) and SI joints (posterior-anterior) were available for all patients.

#### [^18^F]-FDG, [^11^C](*R*)PK11195, and [^18^F]fluoride PET-CT scan

Whole-body PET-CT scans were performed by using a PET-CT type Gemini TF (Philips, Cleveland, OH, USA). Patients fasted for at least 6 hours prior to scanning with [^18^F]FDG. Patients were injected with a mean ± standard deviation (SD) of 111 ± 6 MBq [^18^F]FDG and 375 ± 30 MBq [^11^C](*R*)PK11195, each injection follwed by scanning, with a minimal interval period of 3 hours between the scans. Patients were in a supine position and entered the scanning machine feet first. All PET scans were preceded by a low-dose 35-milliampere-second CT scan covering the vertebral column and the SI joints. Low-dose CT was used for attenuation correction and localization of PET signal and did not allow definite identification of structural bony lesions. Sixty minutes after intravenous injection of [^18^F]FDG and 10 minutes after intravenous injection of [^11^C](*R*)PK11195, whole-body scans of 5 minutes per field of view (FOV) for [^18^F]FDG and 3 to 5 minutes per FOV for [^11^C](*R*)PK11195 were acquired of the total spine and SI joints. The maximum total scan time was approximately 60 to 75 minutes per patient. The total spine and SI joints were depicted in one image. Since the tracer [^11^C](*R*)PK11195 may easily stick to application material, the application system was flushed with 20 mL of NaCl 0.9%, and rest activity was measured after administration of the tracer.

In two additional patients with high disease activity, an extra whole-body (5 minutes per FOV) [^18^F]fluoride PET-CT was performed 1 hour after injection of (mean ± SD) 108 ± 1 MBq [^18^F]fluoride. This PET scan was also preceded by a low-dose 35-milliampere-second CT scan. The application system was flushed with 20 mL of NaCl 0.9%, and rest activity was measured after administration of the tracer.

PET data were normalized and corrected for attenuation, decay, and scatter. All scans were reconstructed as 144 × 144 matrices with a pixel size of 4 × 4 × 4 mm by using a fully three-dimensional line of response iterative reconstruction, including time-of-flight information ('Blob-OS-TF'). These reconstructed images were also used for region-of-interest definition.

#### MRI scan

A gadolinium-enhanced MRI series of the vertebral column and SI joints was performed, preferably within 1 week before or after the PET-CT scans, by using a Siemens Magneton Sonata 1.5 Tesla (Siemens Medical Solutions, Erlangen, Germany). Imaging was done, using the head/neck and spine array, in coronal and sagittal orientation with T1-weighted images before and after gadolinium-contrast plus a short-tau inversion recovery (STIR) sequence. The technical details are as follows: matrix 512 × 512 pixels, FOV of 380 mm in sagittal orientation with slice thicknesses of 3 mm for the cervical/thoracic region and 4 mm for the thoracic/lumbar region (voxel sizes of 0.7 × 0.7 × 3.0 mm and 0.7 × 0.7 × 4.0 mm, respectively). The SI region had a 256 matrix with an FOV of 270 mm in coronal/oblique orientation and a slice thickness of 4 mm (voxel size of 1.1 × 1.1 × 4.0 mm). The T1 post-gadolinium series were made with 0.5 mmol/mL Dotarem(^®^) (Guerbet, France) 0.2 mL/kg, and the total scan time was approximately 60 minutes.

#### Imaging analysis

On conventional radiographs, cervical spine and lumbar spine were visually interpreted and scored according to the m-SASSS scoring method (score of 0 to 72). Additionally, the thoracic spine was scored according to the m-SASSS criteria, although they are not validated for this part of the spine. However, no other scoring methods are available for the thoracic spine. In addition, the involvement of the posterior lesions of the spine was visually determined by one observer and a radiologist. The SI joints were scored according to the modified New York criteria (score of 0 to 8).

A nuclear medicine specialist and a rheumatologist qualitatively interpreted PET-CT data. For relative comparison of tracer uptake, regions of interests were drawn in AMIDE (a Medical Imaging Data Examiner) data analysis software [30]. With the covering low-dose CT as an anatomical reference, elliptic cylinders and rectangular boxes were drawn on top of focal hotspots and a (non-hotspot) vertebral body (in principle, L1, if unaffected) (background), respectively, to calculate mean uptake ratios. MRI data were qualitatively evaluated by a radiologist for subchondral bone marrow edema, gadolinium enhancement, cartilage abnormalities, periarticular erosions, subchondral fatty marrow infiltration, and ankylosis.

### Statistical analysis

Data and images of this pilot study were analyzed in a descriptive manner. Owing to the small study population, a normal distribution of data is not expected. Therefore, patient characteristics were analyzed by using SPSS version 15 (SPSS Inc., Chicago, IL, USA), and values were presented as median (range).

## Results

### Patient characteristics

Baseline characteristics of the included patients are summarized in Table 1.

**Table 1:** Baseline demographic, clinical, functional, and x-ray characteristics of patients

	Low disease activity (BASDAI < 4) (*n *= 5)	High disease activity (BASDAI ≥ 4) (*n *= 7)
Males/females	4/1	3/4
HLA-B27-positive, percentage	100	57
Age, years	31.0 (25-50)	41.0 (24-56)
Duration since diagnosis^a^, years	2.0 (0-4)	3.0 (0-20)
Duration of symptoms^b^, years	12.0 (7-19)	12.0 (1-32)
BASDAI (0-10)	1.7 (0.7-2.2)	6.9 (5.1-9.8)
ESR, mm/hour	14.0 (4-19)	23.0 (2-30)
Sacroiliitis (0-8)	5.0 (4-6)	5.0 (4-7)
m-SASSS (0-72)	2.5 (0-6)	1.6 (0-14)
BASRI-hip (0-8)	1.2 (0-2)	0.75 (0-2)

Values are presented as median (range). ^a^Duration since diagnosis (in years), from definite ankylosing spondylitis (AS) diagnosis according to the modified New York criteria. ^b^Duration of symptoms (in years), from the start of inflammatory back pain. BASDAI, Bath Ankylosing Spondylitis Disease Activity Index; BASRI-hip, Bath Ankylosing Spondylitis Index for the Hip (0 to 4, sum of right and left scores); ESR, erythrocyte sedimentation rate; HLA-B27, human leukocyte antigen-B27; m-SASSS, modified Stoke Ankylosing Spondylitis Spinal Score; Sacroiliitis, sacroiliitis scoring according to the radiographic modified New York criteria (0 to 4, sum of right and left scores).

### Imaging analysis

#### [^18^F]FDG and [^11^C](*R*)PK11195 PET-CT with MRI as a reference

**Figure 1:**
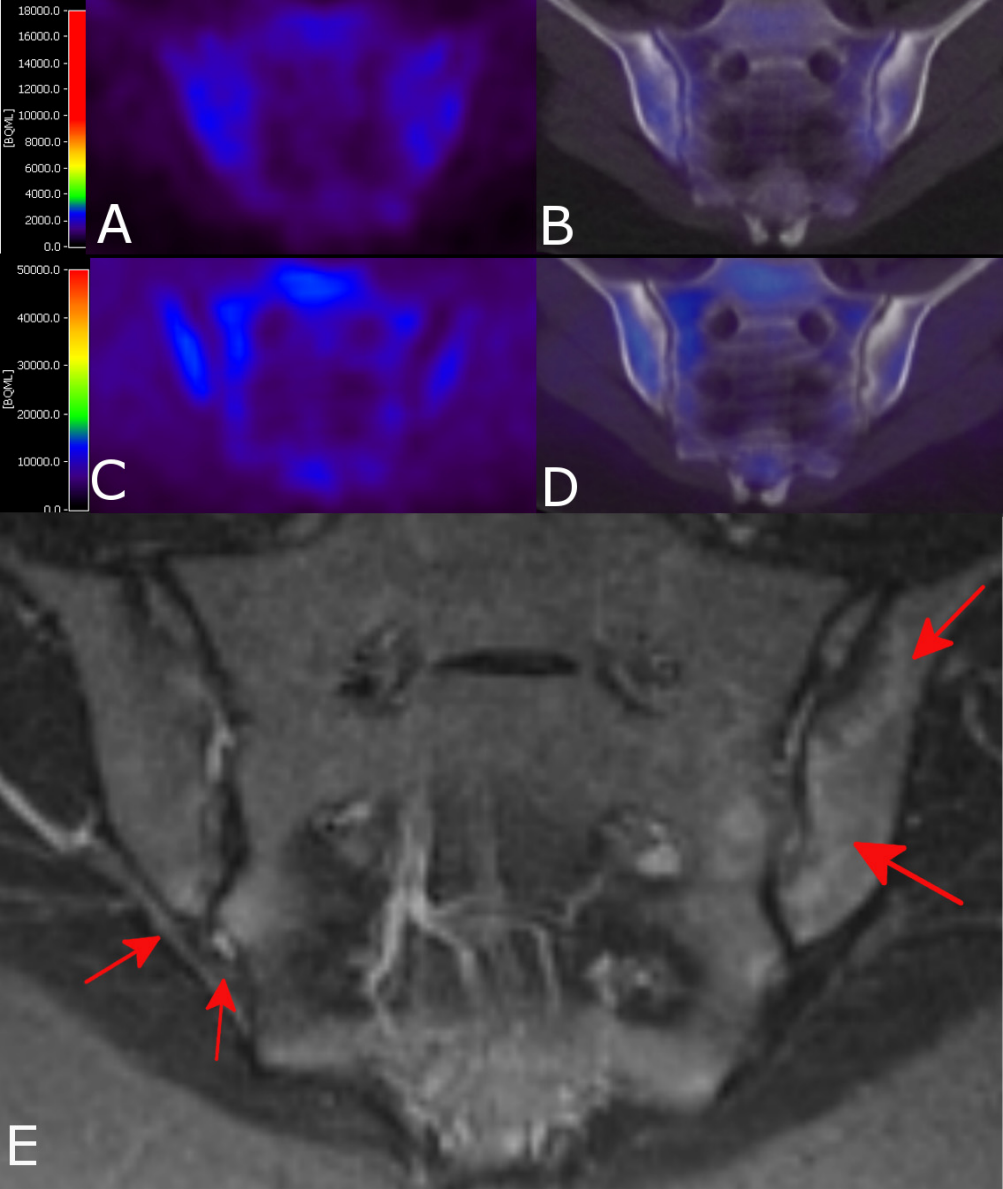
**Positron emission tomography (PET) and magnetic resonance imaging (MRI) images of a patient with high disease activity**. Coronal [^18^F]FDG **(a) **PET and **(b) **PET- computed tomography (PET-CT) and coronal [^11^C](*R*)PK11195 **(c) **PET and **(d) **PET-CT images of sacroiliac joints with no tracer uptake. **(e) **Coronal/oblique MRI (short-tau inversion recovery) of sacroiliac joints of the same patient. Bone marrow edema is present in both sacroiliac joints (indicated by red arrows). [^11^C](*R*)PK11195, PK11195 [(*R*)-1-(2-chlorophenyl)-N-methyl-N(1-methyl-propyl)-3-soquinoline carboxamide]; [^18^F]FDG, [^18^F]-fluoro-2-deoxy-D-glucose.

**Figure 2:**
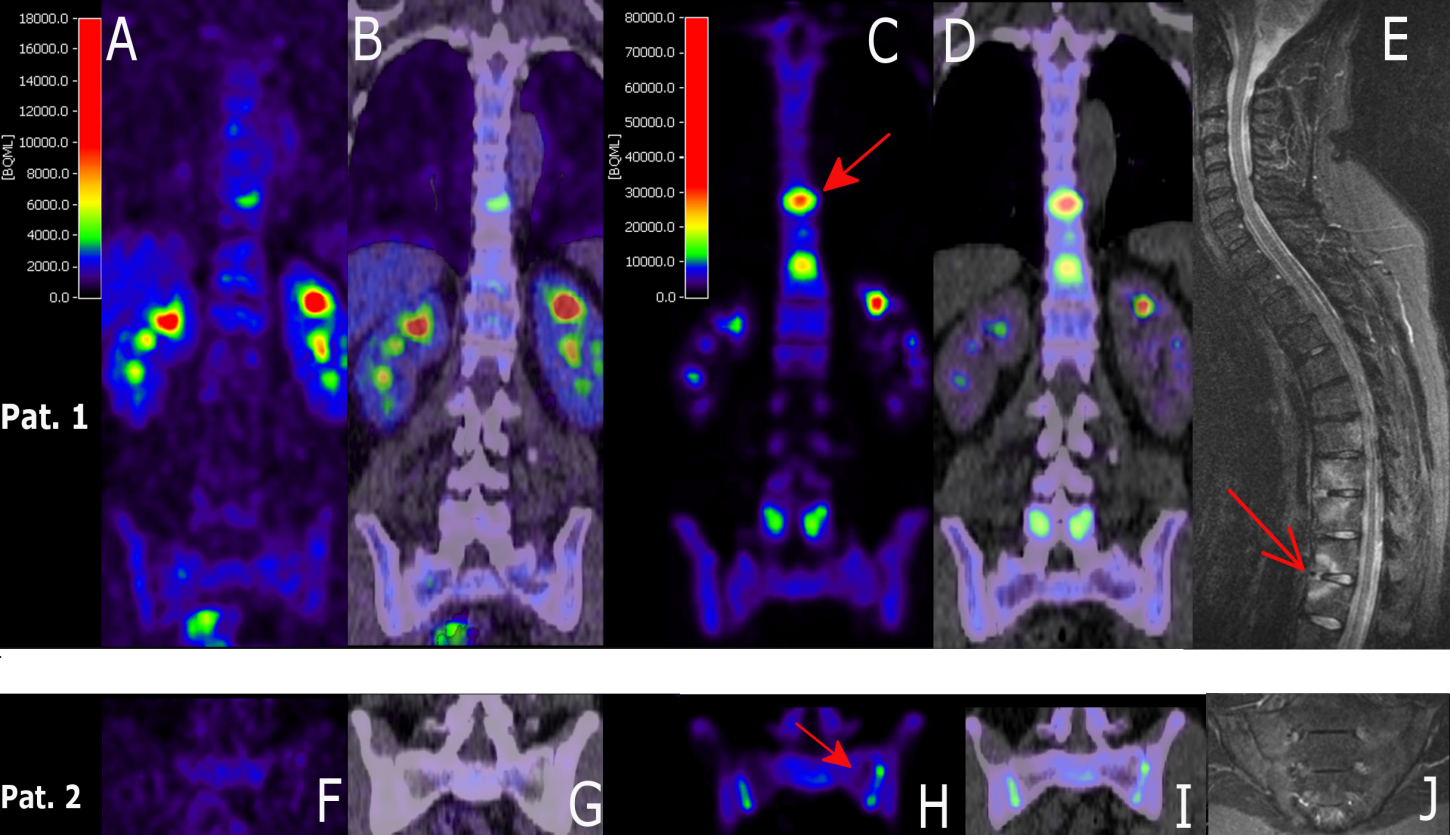
**[^18^F]FDG and [^18^F]fluoride PET-CT and MRI images of patients 1 (a-e) and 2 (f-j)**. Both patients underwent an extra [^18^F]fluoride PET-CT scan. Coronal PET (a, c, f, h) and PET-CT (b, d, g, i) images obtained with [^18^F]FDG (a, b, f, g) and [^18^F]fluoride (c, d, h, i). Multiple hotspots are shown; two examples are indicated by the red closed arrows (c, h). (e) Sagittal MRI image (STIR) of vertebral column and (j) coronal/oblique MRI (STIR) of sacroiliac joints. Multiple lesions with increased signal (bone marrow edema) are shown; an example is indicated by the open arrow. [^18^F]FDG, [^18^F]-fluoro-2-deoxy-D-glucose; MRI, magnetic resonance imaging; PET, positron emission tomography; PET-CT, positron emission tomography-computed tomography; STIR, short-tau inversion recovery.

In none of the first 10 patients was focal [^18^F]FDG or [^11^C](*R*)PK11195 uptake or both noticed (for example, Figure 1c, d). MRI (STIR) images, on the other hand, revealed bone edema (*n *= 5 lesions) in the spine or SI joints of three out of 10 patients (one with low and two with high disease activity) (for example, Figures 1e and 2e). Four out of five lesions on STIR also showed gadolinium enhancement on T1 images. Furthermore, in three patients without bone edema in the SI joints, MRI revealed local ankylosis or subchondral fatty marrow infiltration or both (Figure 2j). Of note, background [^11^C](*R*)PK11195 uptake in bone (marrow) was about five times higher than that of [^18^F]FDG.

#### Comparison of [^18^F]FDG and [^11^C](*R*)PK11195 and [^18^F]fluoride PET-CT with MRI as a reference

In the two additional patients scanned with [^18^F]fluoride, [^18^F]FDG depicted three hotspots in the vertebral column and [^11^C](*R*)PK11195 scans showed no focal uptake. [^18^F]Fluoride scans demonstrated 17 regions with elevated [^18^F]fluoride tracer uptake in both spine and SI joints (Table 2). The three regions revealed by [^18^F]FDG were also depicted with [^18^F]fluoride PET-CT (Figure 2 and Table 2), with an uptake that was five times higher than the [^18^F]FDG uptake.

**Table 2:** Comparison of PET-CT outcome with MRI and conventional radiography of subpopulation scanned with [^18^F]FDG, [^11^C](*R*)PK11195, and [^18^F]fluoride

Patient	Anatomic location		X-ray						**PET-CT**^**a**^				MRI			
			
			**Score**^**b**^		Level		Lesion		[^18^F]FDG		[^18^F]Fluoride		T1+Gd		STIR	
		Cervical	0		2 p		FA		-				-		-	
					3 p		FA									
		
											7 p		7 p		7 p	
					8 a		SC		8a		8 a		8 a		8 a	
					9 a		SC				9 a		9 a		9 a	
1	Spine	Thoracic	0		10 a		SC				10 a		10 a		10 a	
					11 a		SC				11 a		11 a		11 a	
					12 a		SC									
		
		Lumbar	0		4 p		FA		-		4 p		-		-	
					5 p		FA				5 p					
	
	Sacroiliac joints		2	2	R	L	SC	SC								

		Cervical	0		-		-		-		-		6 a		6 a	
													7 a		7 a	
		
											1 p		1 p		1 p	
											6 p					
											8 p					
		Thoracic	0		9 a/p		SQ				9 p					
	Spine				10 a/p		SQ		10a		10 p					
2					11 a/p		SQ		11a		11 p					
					12 a/p		SQ									
		
									-				2 a		2 a	
											3 a					
		Lumbar	2		4 a		SQ									
											5 a					
	
	Sacroiliac joints		4	3	R	L	Ank	SC	-	-	R	L	Ank sfmi	Ank sfmi	Ank sfmi	Ank sfmi

Levels on positron emission tomography-computed tomography (PET-CT) and magnetic resonance imaging (MRI) scans are hotspots with increased signal. ^a^[^11^C](*R*)PK11195 is not presented, because all scans were negative. ^b^X-ray according to modified Stoke Ankylosing Spondylitis Spinal Score (m-SASSS) (0 to 72). a, anterior; Ank, ankylosis; [^11^C](*R*)PK11195, PK11195 [(*R*)-1-(2-chlorophenyl)-N-methyl-N(1-methyl-propyl)-3-soquinoline carboxamide]; FA, facet arthrosis; L, left; p, posterior; R, right; SC, sclerosis; sfmi, subchondral fatty marrow infiltration; SQ, squaring; STIR, short-tau inversion recovery; T1+Gd, gadolinium contrast uptake on T1 images.

MRI depicted nine bone marrow lesions, with enhanced signal on both STIR and gadolinium contrast T1 images, in the spine of these two patients (Table 2). Only one out of three [^18^F]FDG hotspots matched with MRI lesions, and two thirds of the MRI lesions matched with the anatomical position of [^18^F]fluoride uptake. The number of [^18^F]fluoride lesions (*n *= 17) exceeded those detected on MRI (*n *= 9) (Table 2). Eleven active lesions were depicted with [^18^F]fluoride PET-CT and not on MRI. Three lesions (two cervical and one lumbar vertebrae) were detected on MRI and not on [^18^F]fluoride PET. Of note, whole-body [^18^F]fluoride scans additionally showed hotspots in the manubriosternal joints in both patients and in the left acromion-clavicular joint in one patient corresponding to clinical symptoms (data not shown in Table 2).

#### Comparison of [^18^F]fluoride PET and MRI with conventional x-rays

Active sites on [^18^F]fluoride PET-CT or MRI or both were compared with structural changes observed on conventional x-rays (Table 2). In four out of six PET- and MRI-positive lesions, conventional radiography depicted sclerosis. In seven out of 11 PET-positive/MRI-negative lesions, structural changes were found: sclerosis (*n *= 1), squaring (*n *= 3), facet arthrosis (*n *= 2), and ankylosis (*n *= 1). Furthermore, PET or MRI or both depicted nine active sites with no radiographic structural changes. Conversely, seven sites with structural changes on conventional radiography did not depict any activity on either PET or MRI: sclerosis (*n *= 3), squaring (*n *= 2), facet arthrosis (*n *= 2).

## Discussion

Results of this pilot study show the potential of PET-CT imaging of AS activity. Targeting of bone formation appears to be the most promising approach to visualize AS activity. Inflammation tracers ([^18^F]FDG and [^11^C](*R*)PK11195) seem to be less useful for imaging of AS than for imaging of active RA [16,22-24].

There may be several explanations for the discrepancy between [^18^F]FDG, [^11^C](*R*)PK11195, and [^18^F]fluoride PET-CT findings. First of all, since the definite pathogenesis of AS still has to be elucidated, it is unknown which target site for imaging of AS is optimal [18]. Entheses are of special interest, and synovitis seems to be less prominent in AS compared with RA [20]. In addition, syndesmophyte formation and ankylosis, hallmarks of AS, reflect local osteoblastic activity. Our PET-CT findings suggest that bone formation (for example, osteoblastic activity) may be a more prominent feature of AS activity than inflammation. The present PET-CT results with the different tracers, therefore, reveal interesting data that may provide insights into the pathogenesis of AS.

Secondly, tracer biodistribution may be related to varying tracer characteristics. Uptake mechanisms are different for each tracer. In (red) bone marrow, [^11^C](*R*)PK11195 PET scans showed a diffuse increased uptake pattern which could possibly overwhelm potential small focal activity spots in or around bone. [^18^F]FDG is probably a useful marker for synovitis [16,22,23] and osteomyelitis [31,32] but may be less suited for detection of (non- or low-inflammatory) bone formation in AS [33-37]. In general, [^18^F]FDG seems to be more useful for detection of osteolytic than for osteoblastic lesions [38]. [^18^F]Fluoride, on the other hand, reflects osteoblastic activity because of the tracer's uptake into hydroxyapatite crystals, which form the mineral fluoroapatite within bone, especially at sites of bone formation [27,28,39]. Our positive results with [^18^F]fluoride PET-CT in both vertebral column and SI joints of patients with AS correspond with [^18^F]fluoride PET-CT findings of Strobel and colleagues [29] in SI joints of patients with AS and osteo-articular [^18^F]fluoride findings of Ben Ali and colleagues [40], indicating that [^18^F]fluoride is a potential tracer for active bone sites in AS.

A third explanation for the differences in uptake of the three tracers might be related to patient selection. Patients were categorized in low or high disease activity group according to the BASDAI level. BASDAI is a patient questionnaire commonly used to assess disease activity in AS. Congruent imaging results (no active lesions) were found in four out of five patients with low disease activity, corresponding to BASDAI measurements. In contrast, in patients with high disease activity (BASDAI ≥ 4), three out of seven patients with AS did not show any active inflammation on MRI. This corresponds to findings of others who did not find any association between MRI and clinical data [41,42]. Therefore, high BASDAI scores with negative MRI may be more related to secondary degenerative changes and ankylosis rather than active inflammation. This is supported by the finding that six out of seven patients with high disease activity showed bony structural lesions in one or more vertebral bodies; at least 50% of these lesions had degenerative characteristics. Indeed, nowadays, the BASDAI is criticized, and recent studies express the belief that the ankylosing spondylitis disease activity score (ASDAS) is a better selection criterion than BASDAI [43-45]. Furthermore, NSAIDs were continued if used at inclusion. In theory, NSAID use may have suppressed inflammatory activity; however, Gaspersic and colleagues [46] showed that anti-inflammatory drugs have no influence on monitoring AS with MRI. In our study, NSAIDs did not seem to influence [^18^F]fluoride uptake in active lesions since PET-CT scans depicted 17 hotspots in the two patients scanned with this tracer.

PET-CT data were compared with MRI and conventional radiographs as references. Focusing on the two patients additionally scanned with [^18^F]fluoride PET-CT, most MRI lesions corresponded to lesions with [^18^F]fluoride uptake on the PET scan. Additionally, two active [^18^F]fluoride PET-CT lesions in SI joints showed subchondral fatty marrow infiltration on MRI. Although the exact significance of subchondral fatty marrow infiltration is not yet clear, it is believed to be a late structural change due to chronic inflammation in AS [47].

On the other hand, in 11 out of 17 [^18^F]fluoride PET-positive lesions, structural changes were found on conventional x-rays. (Secondary) degenerative changes such as sclerosis and facet arthrosis may result in a positive [^18^F]fluoride signal. However, these lesions could also reflect chronic AS disease activity (the mean symptom duration of patients 11 and 12 was 22 years). Indeed, degenerative changes as well as other structural changes such as squaring and syndesmophyte formation can coexist with chronic inflammation [48]. Finally, the observed [^18^F]fluoride hotspots in the sternum and shoulder seemed to correlate with clinical symptoms of patients, again underlining the potential of [^18^F]fluoride PET-CT to visualize active sites in AS.

## Conclusions

Despite the limited number of patients investigated in this study, our PET-CT results suggest that AS activity was reflected by bone formation and not by inflammation, and this finding throws an interesting light on the pathogenesis of the disease. In addition, [^18^F]fluoride PET-CT seems to be a promising imaging technique for detecting active lesions in the spine and SI joints of patients with AS, but the definite value of [^18^F]fluoride PET-CT as a diagnostic tool for assessment of AS activity needs to be further explored in future studies with larger cohort(s) of patients with AS.

## Abbreviations

AS: ankylosing spondylitis; BASDAI: Bath Ankylosing Spondylitis Disease Activity Index; [^11^C](*R*)PK11195: PK11195 [(*R*)-1-(2-chlorophenyl)-N-methyl-N(1-methyl-propyl)-3-soquinoline carboxamide]; CT: computed tomography; [^18^F]FDG: [^18^F]-fluoro-2-deoxy-D-glucose; FOV: field of view; MRI: magnetic resonance imaging; m-SASSS: modified Stoke Ankylosing Spondylitis Spinal Score; NSAID: non-steroidal anti-inflammatory drug; PET: positron emission tomography; PET-CT: positron emission tomography-computer tomography; RA: rheumatoid arthritis; SD: standard deviation; SI: sacroiliac; STIR: short-tau inversion recovery; TNF: tumor necrosis factor.

## Competing interests

The authors declare that they have no competing interests.

## Authors' contributions

SB made substantial contributions to acquisition, analysis, and interpretation of data and drafting of the manuscript. MvdW made substantial contributions to conception and design of the study, acquisition of data, and drafting of the manuscript. JK made substantial contributions to interpretation of data and revision of the manuscript. OH made substantial contributions to conception and design of the study, analysis and interpretation of data, and revision of the manuscript. RB made substantial contributions to analysis and interpretation of data and revision of the manuscript. JvD made substantial contributions to acquisition of data and revision of the manuscript. BD made substantial contributions to conception and design of the study and revision of the manuscript. AV made substantial contributions to conception and design of the study, analysis and interpretation of data, and drafting of the manuscript. IvdH and CvdL made substantial contributions to conception and design of the study; acquisition, analysis, and interpretation of data; and revision of the manuscript. All authors read and approved the final manuscript.
